# Evaluating nitrogen uptake: Methods and applications for plant research

**DOI:** 10.1002/aps3.70050

**Published:** 2026-03-25

**Authors:** Emilia Pierce, Amanda Rasmussen, Alexandra Huddell, Erin E. Sparks

**Affiliations:** ^1^ Department of Plant and Soil Sciences University of Delaware Newark Delaware USA; ^2^ School of Biosciences University of Nottingham Sutton Bonington LE12 5RD United Kingdom; ^3^ Division of Plant Science and Technology University of Missouri Columbia Missouri USA; ^4^ Donald Danforth Plant Science Center St. Louis Missouri USA

**Keywords:** depletion, isotopes, nanoSIMS, transporters

## Abstract

Nitrogen is an essential nutrient for the growth and development of plants, aiding many physiological and biological functions. Due to the high demand for the nutrient, it is common for agricultural production systems to fertilize crop fields with large quantities of nitrogen. However, excessive fertilization can be harmful economically and environmentally. Understanding the mechanism by which plants take up nitrogen from their environment is critical to optimize plant growth and agricultural productions. Over the past several decades, researchers have used a variety of methods to quantify nitrogen uptake, including using nitrogen isotopes, measuring uptake through depletion of a solution over time, using compartmented chambers or agarose blocks to target specific root regions, and more recent small‐scale approaches such as nanoscale secondary ion mass spectrometry (nanoSIMS), microdialysis, and biomarkers. Several of these studies have been conducted in maize due to its high nitrogen demand and significance in global food and feed production; however, these techniques can be applied to any plant system. This review will examine the application of these methods, highlighting their advantages and limitations. By exploring existing methods, we aim to provide insights into advancing nitrogen uptake studies, ultimately supporting sustainable nitrogen management and improving crop production efficiency.

## Nitrogen fertilization

Plants require 17 nutrients, which can be grouped into two categories: macronutrients (required in large amounts) and micronutrients (required in smaller amounts). Nitrogen, along with potassium and phosphorus, is one of three primary macronutrients. Nitrogen is typically the most limiting nutrient for plant growth and is therefore applied in greater quantities than any other nutrient (Hale et al., 2013). Nitrogen fertilization levels have been rising, with a fourfold increase from 1960 to 2021 (FAO, 2023). However, the excessive use of synthetic nitrogen fertilizer is harmful to the environment, causing acidification of waters and soils, eutrophication of marine ecosystems, biodiversity loss, greenhouse gas emissions, and ozone depletion (Motavalli et al., 2008). Nitrogen fertilizer is also expensive, accounting for 33–44% of maize production costs (Boline, 2025). Despite this intensive input, plants typically take up less than 50% of the applied nitrogen in a given year (Raun and Johnson, 1999; Oenema et al., 2009). Improving our understanding of nitrogen uptake can support the development of crops with enhanced nitrogen use efficiency and enable more precise fertilization strategies, ultimately reducing both the environmental and economic burden of synthetic fertilizer use.

## Nitrogen uptake

Plants can take up dissolved nitrogen in both inorganic and organic forms (Yao et al., 2020). The main inorganic forms of nitrogen are nitrate (NO_3_^−^) and ammonium (NH_4_^+^). Organic sources of nitrogen include urea, amino acids, proteins, nucleic acids, and peptides. Dissolved inorganic nitrogen is preferred for agricultural crops as it is typically more available to plants in agricultural soils (Jones et al., 2005; Moran‐Zuloaga et al., 2015). Although ammonium is the most energy‐efficient form of nitrogen for plants (Abrol, 1990; Courty et al., 2015), nitrate is typically taken up in greater amounts as it is more mobile and offers better availability (Miller and Cramer, 2004).

When a plant root encounters nitrogen, the macronutrient is taken up by specialized transporters (Garnett et al., 2013; Gu et al., 2013; Yu et al., 2019). These transporters are generally specific to nitrogen type and concentration. In maize, nitrate uptake occurs through two main systems. One system, known as the high‐affinity transport system (HATS), operates when roots are exposed to low concentrations of nitrate (<250 µM) and includes transporters in the nitrate transporter (NRT) family NRT2, which have a high affinity for nitrate and a low rate of uptake. This system can be further subset into constitutive (cHATS) and inducible (iHATS) (Crawford and Glass, 1998), representing transporters that are always expressed and those that are induced upon sensing low nitrate concentrations. The other system, the low‐affinity transport system (LATS) operates at higher concentrations of nitrate (>250 µM) and includes transporters in the nitrate transporter 1/peptide transporter family (NPF), which have a low affinity for nitrate and a higher rate of uptake (Abrol, 1990; Glass, 2009). For ammonium acquisition, only HATS has been described, although an ammonium LATS is also likely (Chiasson et al., 2014). These systems work together to optimize nitrate uptake under variable environments.

Of the many forms of organic nitrogen, amino acids are the most researched in terms of plant uptake (Näsholm et al., 2009; Yao et al., 2020). Amino acid transporters fall into two families based on their sequence similarity and uptake properties: the amino acid/auxin permease (AAAP) family, also known as the amino acid transporter family (ATF), and the amino acid–polyamine–organocation (APC) family (Yao et al., 2020). Like inorganic forms of nitrogen, amino acid transporters can also be grouped into HATS and LATS. Although several amino acid transporters have been identified and analyzed, their specific roles are far from understood.

Consistent with the presence of different transport systems, external nitrogen availability influences the nitrogen uptake rate of a plant (Warncke and Barber, 1973; Hole et al., 1990; Raman et al., 1995; Garnett et al., 2013). Due to the sensitivity of these transport systems to external nitrogen concentration, it is common to analyze nitrogen uptake in the context of varying external nitrogen availability. Furthermore, plants that have experienced a period of nitrogen deprivation, followed by a resupply of nitrogen, show an increased rate of nitrogen uptake for a short time period (Hole et al., 1990; Siddiqi et al., 1990; Kronzucker et al., 1995; Gazzarrini et al., 1999; Dechorgnat et al., 2019). Therefore, it is also common to deprive plants of nitrogen before reapplying nitrogen to induce uptake.

Despite a reasonable understanding of the fundamental tenets of nitrogen transport systems, nitrogen uptake remains an understudied area of plant–nutrient interactions due to its complexity and variability. Nitrogen uptake can vary greatly at multiple scales, from whole‐plant differences, driven by species, genotype, or growth conditions, down to root‐level differences, influenced by factors such as root age or root region (Reidenbach and Horst, 1997; Sorgonà et al., 2011). Despite this variability, most research has focused on young, hydroponically grown seedlings for ease of analysis. This limited scope has left gaps in our understanding of nitrogen uptake in various species, environments, and stages of plant development.

Furthermore, many studies fail to connect gene expression of nitrogen transporters with nitrogen uptake. The methods described here have typically been used for general uptake measurements but could also be applied to bridge this knowledge gap by linking transporter activity with nitrogen uptake using nitrogen mutants or pairing uptake studies with transcriptomic studies. The methods described in this review each have their own advantages and disadvantages (Tables 1 and 2), yet they all share the common goal of improving our understanding of nitrogen uptake in plant systems. Continued research focused on improvements to existing measurement methods and development of new methods is crucial to advancing our knowledge of plant nutrition and subsequent agricultural productivity.

**Table 1:** Advantages and disadvantages of microscale nitrogen uptake measurement methods.

Method	Advantages	Disadvantages
^15^N tracer	– Differentiates between nitrogen sources – High accuracy – Relatively inexpensive – Minimal equipment and labor – Suitable for various plant systems and nitrogen forms	– Possible nitrogen efflux from the roots can skew measurements – Isotopic fractionation may cause underestimation – Excision of roots may affect accuracy – Cannot measure uptake in real time unless coupled with another method – Requires isotope‐ratio mass spectrometry (IRMS); results may be delayed
^13^N tracer	– Possibility of real‐time measurements – High precision	– Short half‐life requires local machinery and rapid experiments – Requires expensive equipment – Cannot measure real‐time uptake at the root level of submerged roots
Depletion	– Directly measures nitrogen uptake and rate over time – Compatible with tracers but not necessary – Can provide nearly immediate results – Minimal instrumentation necessary	– High labor and time requirements – Does not show nitrogen uptake location unless coupled with a tracer – Only measures net uptake, not total uptake (includes efflux) – Sensitivity may be an issue with excised roots
Agar	– Measures nitrogen uptake of specific root region – Compatible with tracers but not necessary – Can provide nearly immediate results – Minimal instrumentation necessary	– Cannot perform multiple depletion measurements for time‐course uptake data – Unintended nitrogen losses (e.g., volatilization) may skew results
Compartmented chamber	– Allows in situ measurements – Targeted to specific root regions – Compatible with tracers but not necessary	– Requires young roots with few lateral roots to fit into compartments – Requires compartmented chamber, likely custom built – Potential for tracer leakage across compartments, or efflux out of root into other chambers
NanoSIMS	– Provides high‐precision visualization of ^15^N location after uptake at the subcellular level	– Requires expensive equipment – Intensive sample preparation – Low throughput
Microdialysis	– In situ and minimally destructive – Captures temporal changes in nitrogen availability – Can differentiate between nitrogen form – Isotopes are not necessary	– Cannot measure uptake of nitrogen directly unless coupled with another method

**Table 2:** Advantages and disadvantages of macroscale nitrogen uptake measurement methods.

Method	Advantages	Disadvantages
Sequential harvest	– Does not require manipulations of plants or environment – Does not require expensive equipment – Direct measurement of nitrogen content	– Destructive – Low resolution in time and space – More labor intensive than other macroscale methods
Hyperspectral imaging	– Nondestructive, repeated measurements are possible without plant manipulation – Enables large‐scale/high‐throughput phenotyping	– Relies on chlorophyll content to estimate nitrogen content – Cannot detect overfertilization – Machinery can be expensive – Sensitive to environmental conditions (e.g., light, canopy cover, soil background) – Requires heavy data analysis
Fluorescence sensing	– Nondestructive, repeated measurements are possible without plant manipulation – Enables large‐scale/high‐throughput phenotyping – Rapid, portable, relatively low cost compared to hyperspectral	– Relies on chlorophyll content to estimate nitrogen content – Cannot detect overfertilization – Sensitive to environmental conditions (e.g., light, canopy cover, soil background) – Requires heavy data analysis

## Calculations of nitrogen uptake and kinetics

Nitrogen uptake can be calculated using a variety of kinetic and non‐kinetic methods. Kinetic methods require several substrate concentrations to be tested, while non‐kinetic calculations do not. Examples of non‐kinetic calculations include the macroscale approaches mentioned above, involving the comparison of nitrogen content in plants over time, or remote sensing technologies. Other methods include using isotopically labeled nitrogen to track nitrogen movement or maximum uptake from one high concentration or measuring nitrogen depletion in hydroponic uptake solutions at a single concentration. These methods are helpful to estimate total uptake of plants without needing to manipulate concentrations. However, they do not provide much information about the mechanistic regulation of nitrogen uptake.

Kinetic models, on the other hand, are helpful to analyze how transporter activity can be affected by varying external nitrogen concentrations. Kinetic studies are important for transporter characterization due to the distinct transporter systems, functioning at low or high concentrations. Knowledge of how crops and their transporters function at specific nitrogen concentrations is especially important and applicable when considering fertilization strategies. For example, it may be the case that plants that express more HATS transporters require only minimal fertilizer input, while plants with high expression of LATS transporters may require higher levels of fertilization for optimal growth. Characteristics like these could also be considered in breeding when selecting for plants that function better under certain nitrogen conditions.

Historically, uptake kinetics of nitrogen ions by plant roots has been modeled using the Michaelis–Menten theoretical model of enzyme–substrate saturation kinetics. The model was first used to model root nutrient uptake by Epstein and Hagen (1952) and is described by the following equation:

$$I_{n}=I_{\max}(C-C_{\min})/K_{m}+(C-C_{\min})$$

where *I*_n_ is the uptake or influx rate of the nutrient, *I*_max_ is the maximum uptake rate, *C* is the external concentration of the nutrient in the solution, *C*_min_ is the minimum concentration required for uptake, and *K*_m_ is the binding affinity of the transporter and the ion.

Briefly, the uptake rate or influx (*I*_n_) of the nutrient increases as the external concentration in solution (*C*) increases, until a plateau is reached (*I*_max_). This is when the binding sites of the transporters have been saturated. *K*_m_ represents the binding affinity between the transporter and the ion and is equal to the concentration at half saturation (1/2*I*_max_) (Griffiths and York, 2020). To determine *I*_max_ and *K*_m_, it is necessary to calculate the uptake rate at multiple different concentrations. It is recommended to calculate *I*_n_ over at least 10 different concentrations, ranging from a factor 10 smaller to a factor 10 higher than the expected *K*_m_ (Engels et al., 2000). This process, however, requires substantial time and resources. Therefore, it is more common to determine the maximum uptake rate using one very high concentration of nitrogen.

This model has been significant to our understanding of nutrient transport systems, leading to the original discovery of two separate transport systems functioning at high or low concentrations (HATS and LATS) (Epstein and Hagen, 1952). Although this model is still useful for basic calculations and broad applications, it may not be adequate for in‐depth analysis of transporter activity. The equation assumes that uptake of the entire system of multiple transporters can be represented by one equation; however, this assumption may obscure any unique characteristics of individual transporters in response to nitrogen concentration. For this reason, Griffiths and York (2020) suggest two alternative models: the porter‐diffusion model, used at the cellular level, and the flow‐force interpretation, used at the macroscopic scale (Le Deunff et al., 2016). These models offer more insight into specific transport mechanisms and better capture the dynamic behavior of nitrogen transporters.

## Macroscale vs. microscale approaches to measure nitrogen uptake

A full understanding of plant–nitrogen relationships requires efficient and accurate ways to measure plant nitrogen uptake. Existing methods span from macroscale approaches, such as whole‐plant or population measurements that monitor nitrogen status over time, to microscale techniques that target individual roots or specific nitrogen transporters. While this review will focus on microscale approaches, it is important to be familiar with macroscale methods and how they further our understanding of nitrogen uptake in agricultural systems.

Macroscale approaches can be destructive or nondestructive. An example of a destructive macroscale method is sequential harvest, in which plants are harvested at two different time points and the nitrogen content of the biomass is compared between the two timepoints (Engels et al., 2000). A large increase in nitrogen content over time suggests high nitrogen uptake during that growth period, while a negligible difference in nitrogen content may indicate either limited uptake or saturation. While this method does not require any manipulations of the plants or environment, the results it yields are low resolution in time and space. Additionally, because it is destructive, comparisons must be made between different plants, adding variability and complexity to the interpretation. The method can be modified to include plant or environmental manipulations. However, altering the soil conditions may make it difficult to distinguish whether differences in nitrogen uptake are due to actual plant uptake or changes in nitrogen availability.

Examples of nondestructive macroscale methods include hyperspectral imaging or fluorescence sensing, in which the chlorophyll content of the crop canopy can be detected through imaging equipment such as drones and used as a proxy for leaf nitrogen content. Hyperspectral imaging measures reflected light at specific wavelengths, and fluorescence sensors measure absorbed and re‐emitted light from compounds including chlorophyll. Nitrogen is essential for chlorophyll production, so chlorophyll content is generally well correlated with leaf nitrogen content (Delloye et al., 2018). Nevertheless, several variables including growth stage, cultivars, water, and other nutrient deficiencies may also affect chlorophyll content, limiting the accuracy and comparability of these methods. Furthermore, these methods cannot detect overfertilization due to chlorophyll saturation (Muñoz‐Huerta et al., 2013). Despite these limitations, the minimal manipulation of plants or environments can be advantageous to study nitrogen uptake on a large scale and across time.

Each approach to measure nitrogen uptake involves trade‐offs in terms of cost, precision, real‐world applicability, and disruption to plant systems. Macroscale approaches provide information about real‐world nitrogen uptake but cannot uncover details on specific uptake mechanisms (Table 2). In contrast, microscale methods may reveal details about nitrogen uptake of a specific root, specific root region, or specific transporter, but their results may not be directly representative of nitrogen uptake of the whole plant, or in a real‐world environment (Table 1). Despite this limitation, studying the microscale mechanisms of nitrogen uptake can lead to targeted solutions such as breeding or engineering plants with more efficient nitrogen uptake systems. Once such plants are developed, macroscale techniques can be used to verify the results in a more realistic agricultural setting.

## MICROSCALE NITROGEN UPTAKE METHODS

### The ^15^N tracer method

One of the most common approaches to measure nitrogen uptake is using the stable isotope ^15^N as a tracer. Nitrogen is usually found in the environment as ^14^N, but the stable isotope ^15^N is also naturally present at very low levels, about 0.36% of total nitrogen. When ^14^N is swapped for ^15^N in an uptake solution, the amount of ^15^N taken up by a root can be measured as long as there is enough ^15^N to make the natural background levels negligible. A common application of this method involves placing roots, either excised or intact, into a solution containing at least one ^15^N‐labeled nitrogen source (such as ^15^NO_3_^−^ or ^15^NH_4_^+^) and allowing the plant to take up the nitrogen over a defined time period. This time period might vary depending on the plant, the concentration of nitrogen, or the root type or region that will be analyzed for ^15^N content. This time period must be long enough to allow the roots to take up a measurable amount of nitrogen, but short enough to both minimize any efflux out of the roots and not change expression of transporters in response to the treatment solution. ^15^N uptake studies of roots have used time periods ranging from 10 min (Garnett et al., 2013) to 30 min (Wang and Tsay, 2011; Ishaya and Rasmussen, 2024a), and even 2 h (Rao et al., 1997).

In all cases, roots are removed from the uptake solution and must be rinsed thoroughly to remove excess tracer on the root surface and from the apoplast. Rinsing solutions can be any solution that does not contain the ^15^N tracer. Some reports used distilled water (Rao et al., 1997), while others use CaSO_4_ to help maintain membrane integrity (Minorsky and Spanswick, 1989; Engels et al., 2000; Wang and Tsay, 2011). The rinsing solution could also contain a non‐labeled form of nitrogen at a much higher concentration (10–100‐fold) than what was in the uptake solution (Engels et al., 2000). In other reports, multiple rinses or multiple rinsing solutions are used, often starting with a KNO_3_ rinse to release label binding to the epidermis followed by distilled water rinses (Garnett et al., 2013; Ishaya and Rasmussen, 2024a). It is important to note that for some temperature‐sensitive plants such as maize, a significant decrease in the temperature of the washing solution may cause efflux of the nutrient into the washing solution (Minorsky and Spanswick, 1989). Once rinsed, root tissue samples can be dried, ground, and analyzed for ^15^N content.

Analysis for ^15^N content is usually performed using isotope‐ratio mass spectrometry (IRMS). Briefly, IRMS functions by first combusting the sample, then bombarding the molecules with electrons so that they become ionized. The ions are then passed over a magnet, which will pull lighter ions closer than the heavier ions, thereby separating the ions by weight. It is then possible to calculate a ratio of the different ions (^14^N:^15^N) (Sharp, 2017). The IRMS is typically coupled with an elemental analyzer to measure the total nitrogen content of the sample. Combined, these measurements allow the calculation of the total ^15^N content of the sample.

From the derived measurements of ^15^N content, the Michaelis–Menten equation can be used to calculate uptake rate (*I*_n_) or total nitrogen uptake, the maximum uptake capacity (*I*_max_) of individual roots, and with multiple concentrations, the binding affinity (*K*_m_) (Bassirirad, 2000; Ishaya and Rasmussen, 2024b). This method can also provide insight into nitrogen source preference when different uptake solutions are used, each with a different labeled source of nitrogen (i.e., ammonium, nitrate, amino acid, etc.). In this case, the preferred source can be determined by comparing the uptake rate of the labeled nitrogen source among the different samples. However, this approach is less effective for in situ studies as nitrogen may undergo biogeochemical changes prior to plant uptake. For example, ammonium may be oxidized to nitrate, or an amino acid may be broken down into a form of inorganic nitrogen. Thus, the ^15^N taken up by the plant may not reflect the original form applied. It is also possible to apply ^15^N as a gas to study plant uptake of nitrogen, as has been shown for maize aerial roots that associate with nitrogen‐fixing bacteria (Van Deynze et al., 2018).

The ^15^N tracer also allows for the ability to track the assimilation of nitrogen within a plant. This requires that ^15^N‐fed roots remain intact and experience minimal disturbance. After roots have been fed, tissue samples can be collected from spatially separated regions of the plant (e.g., roots, stems, or leaves) and measured for ^15^N content. It is important to keep the sampling location consistent because ^15^N enrichment can vary both between different tissues and within the same tissue. ^15^N‐enriched tissue samples should be compared with samples from untreated plants to account for any background ^15^N present. It is especially crucial to provide plants with sufficient ^15^N for long enough time periods, because when samples are collected far from the roots, or in plants with large biomass, it is likely that ^15^N will be significantly diluted as it moves through the plant. Furthermore, ^15^N content can be influenced by nitrogen loss processes such as efflux from roots back into the uptake solution, especially when rinsing, or loss through leaves via volatilization. Additionally, it should be considered that ^15^N might not always behave exactly as ^14^N in biological systems (Carlisle et al., 2014), because ^15^N is subject to isotopic fractionation, meaning the lighter ^14^N isotope is preferentially selected over the heavier ^15^N isotope. To account for this possibility, it may be beneficial to have several controls of ^14^N and compare total nitrogen content after uptake to see if fractionation may have occurred. Thus, the ^15^N tracer method may provide direct evidence of uptake and assimilation, but it is also confounded by experimental factors.

### ^15^N uptake in intact vs. excised root systems

To measure ^15^N uptake, it is best if the root remains attached to the plant during ^15^N feeding to provide the most physiologically relevant results. When roots are excised from a plant, nutrient uptake may be disrupted (Engels et al., 2000; Falkengren‐Grerup et al., 2000; Brackin et al., 2015). Although uptake of excised roots may not directly represent real‐world conditions, measurements can still be useful for certain aims such as comparison between root types, genotypes, or characterizing the activity of specific membrane‐bound nitrogen transporters (Garnett et al., 2013; Gu et al., 2013; Ishaya and Rasmussen, 2024b). Alternatively, intact root systems provide results that are more reflective of real‐world conditions but can be influenced by several factors that can also hinder the accuracy of uptake results. For example, it is common to use intact roots of hydroponically grown plants, but their root development and nutrient uptake are not representative of soil‐grown plants. If plants are grown in the field, it is possible that the roots may get damaged when the plant is excavated from the soil for testing, and any mycorrhizal connections will also be broken, further removing any real‐world application. To prevent this, plants can be grown in rhizoboxes or in an apparatus that allows for easy access to the roots with minimal disturbance. It is important to note that plants grown in these conditions will likely have altered root structures compared to field‐grown plants and might experience differences in nutrient availability due to the controlled growth conditions, which could affect nitrogen uptake rates.

To avoid these drawbacks, ^15^N uptake studies can also be done on undisturbed in situ field‐grown plants. Although this focuses more on nitrogen assimilation rather than uptake of specific roots, it reduces damage to roots while maintaining natural growing conditions. To measure ^15^N uptake of in situ field‐grown plants, ^15^N can be poured onto the soil surface (Vos et al., 1993; Gardner and Drinkwater, 2009) or into trenches near the plant (Ishaya and Rasmussen, 2024b), and accumulation within the plant can be measured. This results in minimal to no damage to the plant or its root system, but it can be difficult to control the concentration of ^15^N directly available to plant roots. Furthermore, this jeopardizes any future experiments conducted in the same field due to the manipulation in the amount of ^15^N in the soil.

### The ^13^N tracer method

An alternative nitrogen isotope tracer method involves the use of the ^13^N radioactive isotope. When a positron (such as ^13^N) decays, it emits two γ rays in opposite directions (Kiyomiya et al., 2001). These rays can be measured by either a liquid scintillation counter (although this does not allow for real‐time analysis) or a measurement system such as the positron‐emitting tracer‐imaging system (PETIS) developed by Hamamatsu Photonics of Japan and the Takasaki Ion Accelerators for Advanced Radiation Application (TIARA) group (Matsunami et al., 1999; Kiyomiya et al., 2001). PETIS uses a scintillation camera to detect the γ rays, providing the ability to see real‐time movement of elements in a two‐dimensional space (Kiyomiya et al., 2001). In an alternative analysis approach, positron emission tomography (PET) imaging technology allows for nondestructive spatial and kinetic measurement of a radio‐tagged molecule (such as ^13^N) within a living subject over short periods of time in a three‐dimensional space (Kronzucker et al., 1995; Komarov and Tai, 2022).

**Figure 1:**
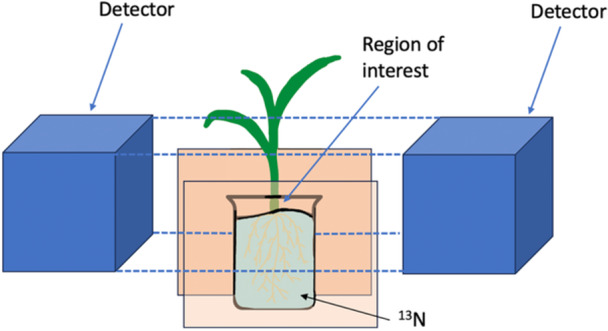
Simplified diagram of a potential ^13^N uptake experimental setup depicting a plant placed in an uptake solution containing ^13^N, between two positron‐emitting tracer‐imaging system (PETIS) detectors that measure ^13^N uptake at the basal region of the plant.

Positrons, including ^13^N, are made using a cyclotron. Due to the short half‐life of ^13^N (9.97 min), local access to a cyclotron is required. ^13^N is typically created via proton irradiation of H_2_O, which produces primarily nitrate (^13^NO_3_^−^). If desired, this can be converted to ammonium (^13^NH_4_^+^) using a chemical process described in Kronzucker et al. (1995). Measuring nitrogen uptake using the ^13^N tracer is usually done with intact roots of young, hydroponically grown plants. The roots are grown in an unlabeled nutrient solution and are transferred to a solution containing ^13^N immediately before measurement. Kiyomiya et al. (2001) used a PETIS detector to study ^13^NH_4_^+^ uptake and movement in rice roots. They placed a single plant in a polyethylene bag containing an uptake solution without any ammonium and balanced the bag between two boards between the PETIS detectors, which were pointed at the basal part of the shoot or the leaves (Figure 1). They then added ^13^NH_4_^+^ to the uptake solution, and the emitted γ rays were counted by the detectors for 60 min. The plant was then removed from the uptake solution, the roots were rinsed to remove excess ^13^NH_4_^+^, and the plant was placed in a Bioimaging Analyzer System (BAS) imaging plate (BAS‐1500 Imaging System; Fujifilm, Tokyo, Japan) for 10 to 20 min.

The ^13^N tracer method can also be used to calculate total nitrogen uptake amount, uptake rates in real time, and where the nitrogen travels within the plant after uptake. To measure total nitrogen uptake, plants are placed in the solution containing ^13^N for a set amount of time, removed, washed with normal ^14^N (to desorb any remaining ^13^N from the surface), and the amount of ^13^N in the root is calculated. Real‐time measurements of nitrogen uptake rates can be measured using scintillation imaging as described in Kiyomiya et al. (2001). Heat maps can be produced to show where in the plant the ^13^N traveled, as well as its concentration. Results can also be presented in a numerical format, allowing the accumulation of radioactivity to be plotted over time.

Although many studies using ^13^N to investigate uptake were performed over 20 years ago, recent articles (Mincke et al., 2021; Komarov and Tai, 2022) have described the promise of PET for plant research due to an increase in popularity of the imaging technology in clinical research. It is predicted that PET machinery will be more common, and therefore more ubiquitous and accessible for plant research. However, access to a local cyclotron to produce the ^13^N will continue to be a limiting factor for these analyses.

### Depletion

An alternative to measuring uptake with isotopic nitrogen sources is measuring uptake through depletion. The depletion method measures nitrogen uptake by monitoring the decrease of nitrogen concentration in an uptake solution over time. The difference between the initial and final concentration of nitrogen is attributed to the total net root uptake. These measurements can be used to calculate net nitrogen uptake rates and total nitrogen uptake capacity. While labeled nitrogen tracers are not necessary for this method, ^15^N can be used for the added benefit of determining the translocation of nitrogen within the plant after uptake.

Jia et al. (2023) used this method to determine the nitrogen uptake rate of maize seedlings. Seedlings were grown in a nutrient solution containing nitrate and placed in a nitrogen starvation treatment 96 h before experimentation. Intact roots were then placed in an uptake solution containing nitrate. Root and solution samples were collected at various time intervals, the fresh weight of the root was recorded, and NO_3_^−^ in solution was measured using the sulfuric acid–salicylic acid method (Cataldo et al., 1975) at 410 nm. This works by reacting nitrate with salicylic acid in sulfuric acid to form nitrosalicylic acid, which when mixed with a base such as NaOH turns yellow and can be quantified by measuring the OD_410_ value. In this study, nitrogen in solution was sampled only once per root at the time of collection (Zhao and Wang, 2017). Alternatively, nitrogen in solution can be measured repeatedly for the same root over time. For example, Sorgonà et al. (2011) exposed roots to a nutrient solution containing 50 μM KNO_3_^−^, took a 100 μL solution sample every 10 min throughout a 60‐min period, and measured the nitrate concentration at 210 nm using an ultraviolet–visible (UV‐Vis) spectrophotometer. UV‐Vis spectrophotometers operate by passing light in the ultraviolet to visible light range across a sample and measuring the light absorbed (Agilent Technologies, 2025). Nitrate can be quantified in this way as it has strong absorbance around 210 nm (Maguire et al., 2022).

A high‐throughput phenotyping pipeline called RhizoFlux has also been developed to phenotype multiple ion‐uptake kinetics simultaneously (Griffiths et al., 2021). RhizoFlux utilizes the depletion method, in which several plants are grown in individual hydroponic chambers and the net nutrient uptake rate for specific nutrients is determined by measuring the concentration of the nutrient in the depleted solution over time using ion chromatography. Using RhizoFlux, researchers were able to quantify specific uptake rates of different nutrients simultaneously. This allows investigation into the interplay of multiple nutrients, which is more representative of field conditions where plants are exposed to multiple nutrients simultaneously. However, Rhizoflux cannot fully replicate the complex root–soil interactions that occur in field conditions, and so results should be interpreted with caution.

Depletion studies can be done on entire root systems, individual roots, or even specific regions of individual roots. The depletion method can also be used in situ with larger roots from mature plants, especially those, such as trees, that cannot be easily grown in lab settings. McFarlane and Yanai (2006) studied several different variations of the depletion method to measure nitrogen uptake in intact roots of mature trees. There are several variations of the depletion method (see below, under “Agarose‐block method” and “Compartmented container”) that can be used or adjusted as necessary.

#### Agarose‐block method

This method is a variation of the depletion method that involves measuring the depletion of nitrogen from a block of agarose applied exogenously to roots. Nitrogen (labeled or unlabeled) is dissolved in an agar mixture and left to set. A block of agar of known size and volume is then directly applied to specific roots or root regions of interest. After the agar has been applied for a user‐defined amount of time, the agar is removed from the root, boiled to dissolve, and the amount of nitrogen remaining in the dissolved solution is quantified (Reidenbach and Horst, 1997). Using a nitrogen tracer can provide similar information and has the added advantage of determining assimilation patterns within the plant. A control should also be implemented in which a block of the nitrogen containing agar is left in the experimental location without plant interaction for the duration of the uptake experiment. Reactions such as volatilization, precipitation, or leaching can occur that might decrease nitrogen content in the agar over time independent of plant uptake (Engels et al., 2000).

Reidenbach and Horst (1997) used this method to measure NO_3_^−^ uptake of different root regions of maize plants grown both in a nutrient solution and in rhizoboxes. To determine uptake of hydroponic plants, maize plants were grown in solution with 1 mM nitrate for 12 days, then placed in a solution without nitrate for two days. The primary root was then covered with agarose blocks containing 1 mM nitrate. After 8 h, the blocks were removed, boiled in distilled water, centrifuged, and the NO_3_^−^ content of the supernatant was determined. The nitrate uptake rate was calculated based on the depletion of the agarose blocks and the surface area of the respective root segments. Uptake of roots grown in rhizoboxes was measured in a similar manner. Accessible roots (roots growing along the observation glass) were covered for 6 h with agarose blocks with 1 mM nitrate. After 6 h, the agar was removed and NO_3_^−^ content was determined as previously described.

#### Compartmented container

**Figure 2:**
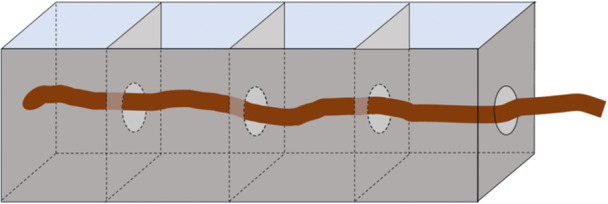
Diagram illustrating a potential compartmented chamber setup, where a root is threaded through openings between separate compartments. These openings are then sealed, and each compartment can be filled with the desired uptake solution.

The compartmented container method is another variation of the depletion method that, like the agarose block method, can measure nitrogen uptake of specific root regions. Unlike the agarose block method, this approach measures uptake across a three‐dimensional section of a root rather than uptake of a two‐dimensional surface area. Briefly, a container with a series of compartments connected by small holes is filled with soil or nutrient solution. A root is woven through the holes on the side of each compartment, so that it passes through each of the compartments (Figure 2). Each hole is sealed with a substance such as petroleum jelly or silicone around the root so that uptake solution cannot travel between compartments. Uptake can then be measured from a selected portion of the root either by measuring nutrient depletion from a selected compartment (Sorgonà et al., 2011), or by applying a tracer to one compartment and measuring accumulation within the plant (Drew and Saker, 1986). This method is commonly done with young, hydroponically grown plants. Sorgonà et al. (2011) measured the spatial variability of nitrate uptake along the maize primary root axis by placing the primary root of a 7‐day‐old maize seedling into a container with five compartments each measuring 2 × 2 cm. The root was woven through 3‐mm holes in the sides of each compartment and sealed with silicone. Next, the root was pretreated with an aerated nutrient solution containing 50 µm KNO_3_ for 0, 4, 6, 8, and 24 h. At the beginning of the uptake assay, the nutrient solution was replaced with 1 mL of the same solution and samples (100 µL) were collected every 10 min over a 60‐min period; NO_3 _^−^ concentration was then measured at 210 nm using a UV‐Vis spectrophotometer. This method can also be performed in the field by training roots to grow into bags or trays, or by gently excavating roots near the soil surface (McFarlane and Yanai, 2006).

### NanoSIMS

As described above, stable isotopes like ^15^N are useful for monitoring nitrogen uptake or translocation patterns at the scale of whole tissues. These techniques are useful for whole‐root‐scale or whole‐plant‐scale information and allow somewhat large‐scale high‐throughput analysis; however, they lack the detailed spatial information needed to study processes on the microscopic cellular or subcellular scale. This is where techniques like secondary ion mass spectrometry (SIMS) are useful. SIMS allows imaging of ionic distributions within a tissue, including differentiating between different isotopes (like ^15^N and ^14^N).

SIMS analysis occurs under a high vacuum where a beam of ions bombards a surface with heavy/energetic ions, which then scatter ions on the surface (secondary ions). Secondary ions with slightly different masses will react differently, resulting in different scattering patterns (Moore et al., 2012). When the beam of ions is scanned across the tissue (e.g., a leaf), the resulting scatter is recorded at each location, creating an image of where particular ions were located. If ^15^N was measured in that scatter, the spatial location of the ion across the leaf can be determined.

There are two SIMS types: static and dynamic. Briefly, static SIMS uses lower energy and can identify large molecules (masses from 1 to >10,000 Da) because the low‐energy beam leaves the compounds intact (while higher‐energy beam techniques split compounds into smaller components), but sacrifices spatial resolution. In contrast, dynamic SIMS uses a higher‐energy beam of ions to blast the surface. This can be accurate on the nanoscale (and hence used for nanoSIMS) but can only be used for small or atomic ions such as nitrogen (Moore et al., 2012).

The analysis of ionic responses of surfaces at the nanoscale is referred to as nanoSIMS, and resolution can be as fine as 50 nm (for negative ions) and 150 nm (for positive ions) (Moore et al., 2012; Kopittke et al., 2020). The difference in spatial resolution between negative and positive ion detection is related to the ion beam energy, as cesium beams are used to produce negative ions and oxygen beams are used to produce positive ions. Oxygen beams are higher energy than cesium beams, and they trigger a wide range of secondary positive ion scatter that requires further filtering, reducing the resolution compared to that achieved for negative ions. Because of this fine resolution, nanoSIMS has been used to study nitrogen (and carbon) dynamics in the root rhizosphere (Jones et al., 2013), soil‐type‐specific organic matter breakdown (Kopittke et al., 2018), nitrogen and carbon exchange between fungi and root cells within a mycorrhizal root tip (Mayerhofer et al., 2021), and patterns of nitrogen broken down by microbes from plants (Schmidt et al., 2023) or by endophytic or leaf‐associate microbe populations (Tarquinio et al., 2018; Sher et al., 2024).

Because nanoSIMS analysis occurs under high vacuum, it is important to remove the water from the samples (Moore et al., 2012). To achieve this, samples are usually frozen and then dehydrated, or alternatively, they are fixed, dehydrated, and embedded in resin (Moore et al., 2012; Jones et al., 2013). While the resolution of nanoSIMS has huge potential to provide insight into processes at this fine scale, the preparation and time required for nanoSIMS preclude use of the technique for large sample sizes, as used in the techniques described above. As always, the right technique will depend on the research question being addressed.

### Microdialysis

Microdialysis has been used extensively in neurobiology (Bungay et al., 1990; Saylor and Lunte, 2015) but has more recently been adopted for soil sampling applications (Inselsbacher et al., 2011, 2014; Oyewole et al., 2014). The exact applications of microdialysis vary depending on what analysis technique it is coupled with, but it can be used to monitor nutrient patterns, processes such as organic and inorganic nitrogen patterns in different ecosystems, proximity to plant roots, or comparing soil types (see below for other examples). The method relies on ions passing from the soil, across a semi‐permeable membrane, and down concentration gradients into a sampling tube. The collected solution containing soil‐derived ions can then be analyzed using methods such as colorimetric reactions or ultra‐performance liquid chromatography (UPLC) quantification of molecules. UPLC functions by separating a wide range of small molecules and ions, whereas colorimetric methods are reaction‐based, providing quantification of only the molecule involved in the reaction. UPLC also incorporates high‐precision detectors, which provide higher quantification accuracy than the colorimetric methods; moreover, it is a minimally invasive method and removes only dissolved compounds but not soil water from the surrounding area. It is important to note that microdialysis does not directly measure nitrogen uptake by the plant but instead measures the available nitrogen in the soil—similar to the depletion method described above but with higher spatial resolution. This may provide insight into available nutrient concentration, mobility, and turnover rates in the field, and does so in nearly real time. In soil, the method has proven able to measure trace metals, chloride, nitrogen, and low‐molecular‐weight organic anions (Miró and Frenzel, 2003; Miró et al., 2005; Rosende et al., 2013; Cocovi‐Solberg et al., 2014; Inselsbacher et al., 2014) and, when combined with three‐dimensional micro‐computed tomography (microCT) imaging, can provide nutrient information about locations with known soil structures or known distances from roots (Brackin et al., 2017). Although it does not measure plant nitrogen uptake directly, it can be paired with the uptake methods described above for a more robust overview of plant–nitrogen relations.

## DISCUSSION

**Figure 3:**
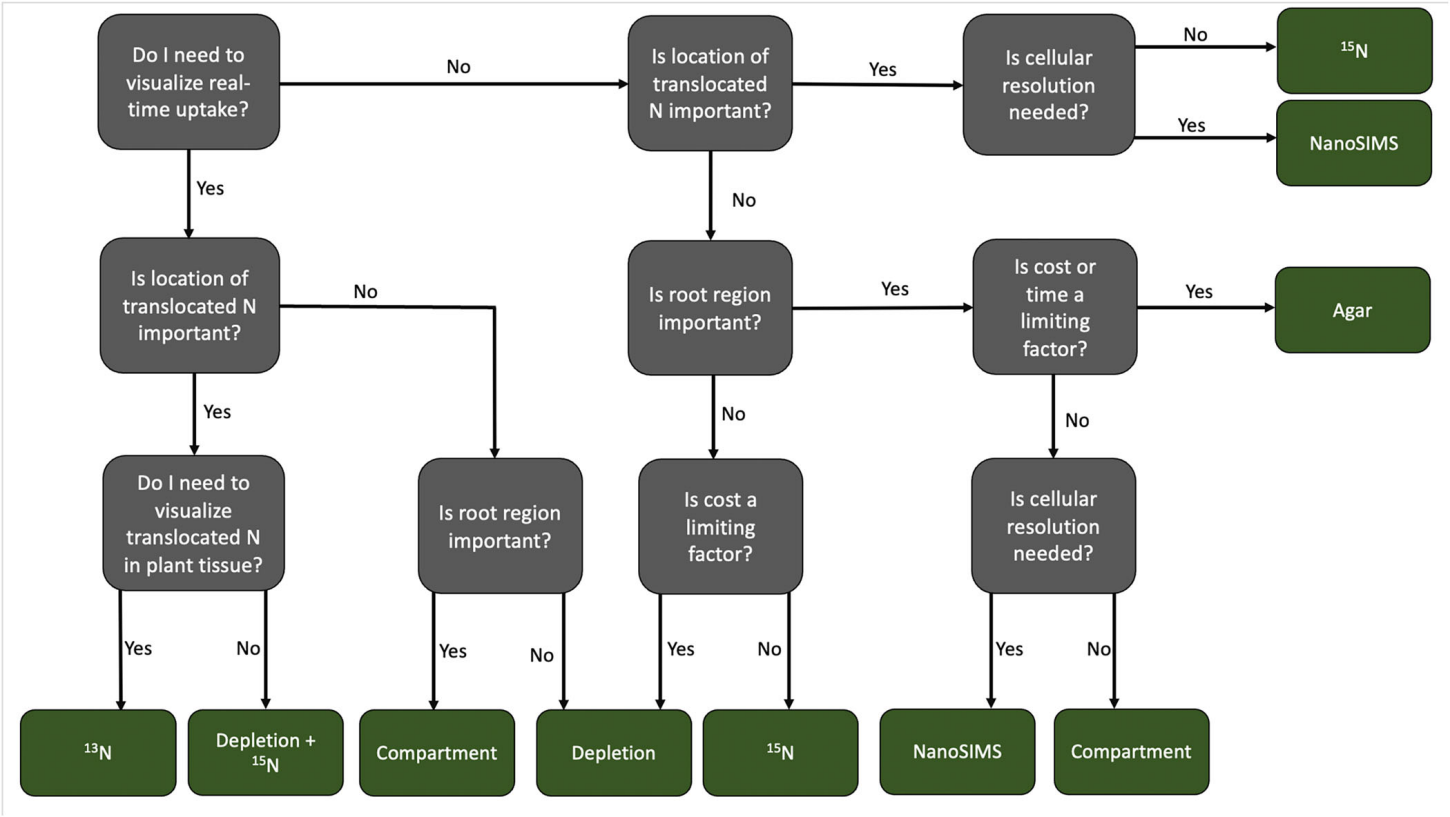
Decision tree to determine optimal nitrogen uptake measurement based on experimental goals.

Although nitrogen uptake has been studied for several decades in many plant systems, there are still several critical knowledge gaps and opportunities to improve our understanding of nitrogen uptake in plants. Awareness of the currently used methods allows researchers to select the best method for their experimental question (Figure 3), furthering our understanding of plant–nitrogen interactions. A comprehensive comparison of the existing nitrogen uptake measurement methods is needed to validate the accuracy of uptake measurements, as well as to identify inaccuracies, ultimately leading to improvements of these methods. Future research should focus on comparing precision‐focused techniques, such as PET imaging, with more scalable and practical approaches (e.g., depletion). Balancing precision, scalability, and relevance to field applications will be necessary to advance our understanding of nitrogen uptake and its practical implications for sustainable agriculture.

Variations within methods should also be investigated. Standardizing key variables such as the length of exposure to the uptake solution, nitrogen concentrations, rinsing protocols, and the inclusion of CaSO_4_ would allow easier comparisons between studies. This would provide a more holistic understanding of nitrogen uptake, both in a specific crop and between different crops. Furthermore, some of these variables could still benefit from more research. For example, some rinsing steps to remove ^15^N tracers may either leave behind unabsorbed nitrogen in the apoplast or on the root surface or may trigger significant efflux. More research is needed to determine optimal rinsing protocols that balance these trade‐offs and to assess whether rinses sufficiently clear the apoplastic space of the tracer, while limiting significant efflux.

Recent efforts to study nitrogen uptake mechanisms in maize have been increasingly focused on nitrogen transporters (Quaggiotti et al., 2004; Trevisan et al., 2008; Gu et al., 2013; Garnett et al., 2013, 2015; Dechorgnat et al., 2019), yet transporter‐level uptake kinetics are still poorly understood (Griffiths and York, 2020). While many studies performed in maize have identified relationships between the gene expression of nitrogen transporters and uptake rates (Quaggiotti et al., 2004; Trevisan et al., 2008; Garnett et al., 2013, 2015; Lupini et al., 2016), they have not investigated transport kinetics such as *I*_max_ or *K*_m_. Integrating uptake measurement techniques with transcriptomic data and machine learning could strengthen this relationship, leading to a better understanding of nitrogen uptake mechanisms. Furthermore, a relationship between transcript abundance and transporter density has not been investigated (Griffiths and York, 2020). Although it has been predicted that a higher transcript abundance will result in higher transporter density, resulting in higher uptake levels, this has not been confirmed. In addition to the gene expression of nitrogen transporters, root system architecture also plays a role in nitrogen uptake. Therefore, a physical model that considers both root system architecture and transporter dynamics would be beneficial and provide more information than kinetic modeling alone.

On a larger scale, the use of nitrogen‐responsive genes as biomarkers to determine plant nitrogen status has shown promise. Yang et al. (2011) demonstrated that the expression profiles of a subset of nitrogen‐responsive genes are proportional to the nitrogen status of a plant. The authors were able to use these biomarker genes to monitor nitrogen status nondestructively in field‐grown plants. This approach might help bridge the gap between lab‐based studies and field‐based research. Biomarker assays may also be a good predictive technology with utility for seed selection and breeding or for assessment of mid‐season nitrogen status. However, this method has not been widely adopted.

It is important to note that the microscale measurement techniques described in this review determine nitrogen uptake rates under idealized lab conditions. The ultimate objective, however, is to better understand and eventually enhance nitrogen uptake in field settings. This future research could eventually enable the design of nitrogen uptake systems optimized for specific crops that could consider environmental variables like nitrogen availability, precipitation, temperature, and light. Research is needed to bridge the gap between laboratory and field conditions by assessing how nitrogen uptake differs between these environments. This could involve integrating macroscale techniques or developing new approaches to measure nitrogen dynamics in realistic, field‐based scenarios. Advancements such as this will be vital for improving nitrogen use efficiency in agriculture and supporting sustainable crop production.

## AUTHOR CONTRIBUTIONS

E.P. wrote the initial draft. A.R. and E.E.S. acquired the funding. All authors conceived the research topic, revised the manuscript, and approved the final version of the manuscript.

## Data Availability

No original datasets were used in the preparation of this review.
